# A special issue of *Essays in Biochemistry* on the social lives of bacteria

**DOI:** 10.1042/EBC20260070

**Published:** 2026-09-02

**Authors:** Freya Harrison, Rachel M. Wheatley

**Affiliations:** 1 School of Life Sciences, University of Warwick, Coventry, U.K.; 2 School of Biological Sciences, Queen's University Belfast, Balfast, U.K.

**Keywords:** competition, cooperation, microbial ecology, social evolution

## Abstract

The study of bacterial sociality, understanding when and how bacteria interact and the consequences of those interactions, has rapidly grown in popularity over the last 20 years. This has revealed that bacteria engage in a remarkable variety of social interactions, including complex cell–cell communication, the exchange of an arsenal of inhibitory molecules, and the sharing of metabolites. These interactions can have significant consequences for the host or environment they reside in, and numerous mechanisms of interaction have attracted significant interest for their therapeutic potential. The present special issue contains eight review articles addressing three major questions in bacterial sociality (*Who is where? How are they interacting? And what are the implications of this?*) alongside five methods-focused articles that introduce some particularly valuable approaches for the study of bacterial interactions.

Bacterial sociality was something of a niche topic when we began our scientific careers, but the community of researchers working on how microbes interact and communicate, and how this influences their ecology, evolution and ecosystem impacts, appears to have undergone a population explosion in the early 21st century (Figure 1). At the same time, advances in imaging, sequencing technologies, and computing have allowed us to investigate the biogeography and evolution of bacterial populations and communities in precise detail. The study of bacterial social interactions is now considered a key subfield of microbial ecology and evolutionary biology. Its importance is also increasingly recognised within applied and clinical microbiology (e.g. [1].). Some important advances include understanding that biofilms exist as the natural state of most bacterial communities [2], appreciation of the extent of direct competition (e.g. killing or inhibition systems) that exists between bacteria [3], and determining the mechanisms by which microbiome diversity can protect against pathogen infection [4]. Within this space, we can define three major questions: (1) Which microbes are where? (2) Who are they interacting with and how? (3) What are the implications of this [1]?.

**Figure 1:**
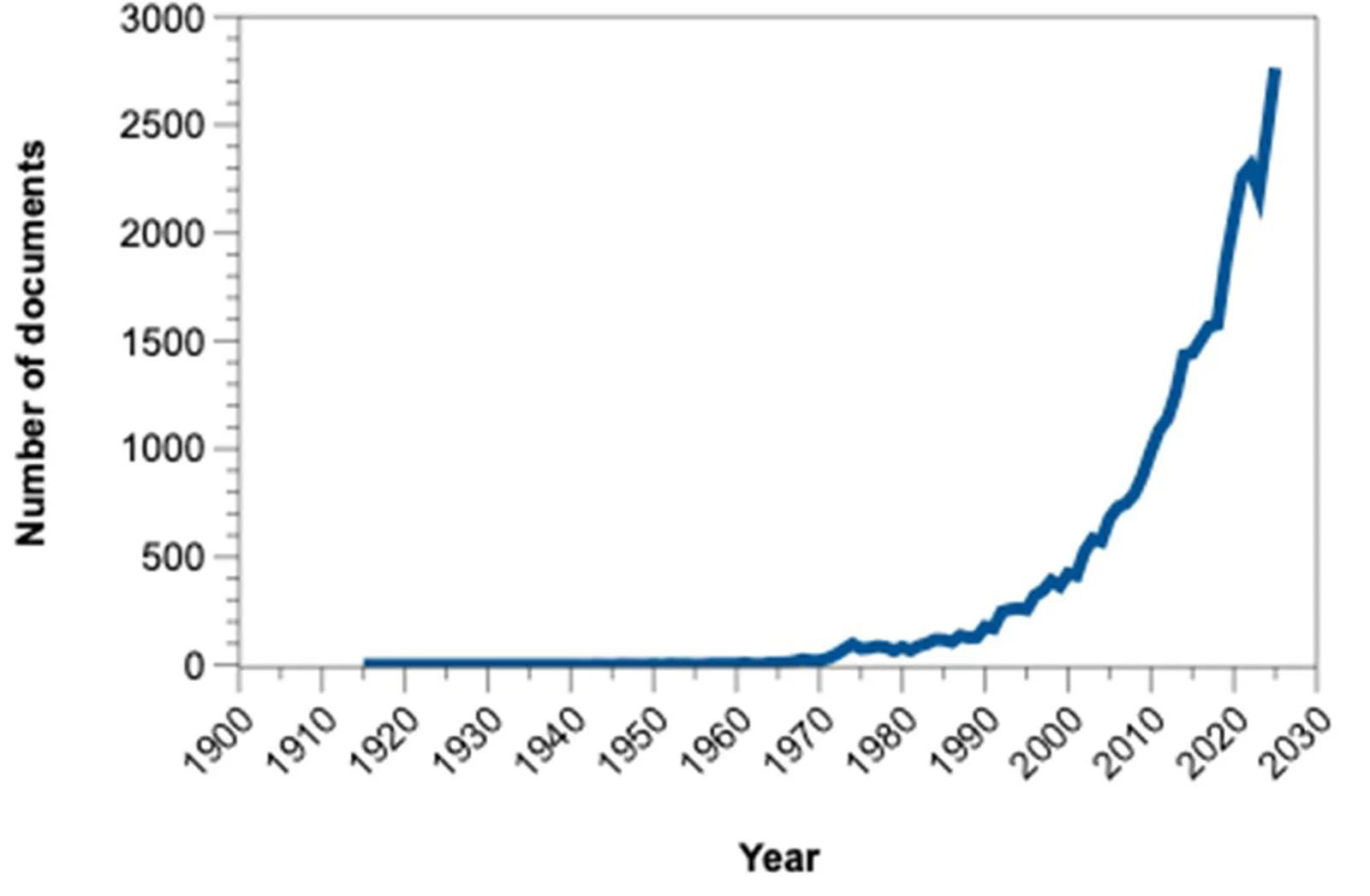
Annual number of published research articles and reviews pertaining to bacterial sociality, as indexed in Scopus As a rough gauge of research interest in the social lives of bacteria, we searched scopus.com on June 24th, 2026 for records whose title, abstract, or keywords contained either ‘bacteria’ or ‘bacterial’ and any of ‘social,’ ‘cooperation,’ or ‘competition.’ A total of 38,932 documents were returned with publication years up to and including 2025. A further 1,569 papers had already been published in 2026.

The present special issue celebrates the rise of bacterial sociality as a research field over the last 20 years, outlines some of the key research advances in this space, and highlights future promise of this field to tackle important challenges of the 21st century, such as combatting antibiotic-resistant pathogens or improving plant health and resilience. The articles published in the present special issue reflect review topics spanning the breadth of these three major questions (*Who is where? How are they interacting? What are the implications of this?*). The present issue also includes five methods-focused articles that are designed to introduce the reader to especially useful approaches for the study of microbial interactions. In each case, the expert authors provide both a practical overview of how to apply this approach and a critique of its strengths and limitations.

## Who is there?

In a complementary set of methods-focussed papers, Baxter et al. [5], Bakker [6], Mark Welch [7] and Minero et al. [8] present reviews of four advanced imaging modalities that allow researchers to visualise microbial interactions at a range of scales, each allowing the exploration of different features of microbes and their communities. Baxter et al. describe the ability of MesoLens to combine sub-micrometre resolution with a multi-millimetre field of view to visualise unusually large areas of bacterial communities such as biofilms [5]; at the other end of the scale, Bakker introduces the application of cryo-scanning electron microscopy to reveal near native-state biofilm matrix at the nanometre scale [6]. Rapid freezing allows preservation of the highly hydrated biofilm matrix during imaging, revealing details of interactions between cells, matrix, and growth substrate. Mark Welch [7] and Minero et al. [8] focus on fluorescence microscopy and how judicious choice of labels and application of computing power can allow the visualisation of multiple cohabiting microbial taxa (CLASI-FISH) or the identification of multiple biofilm matrix components, respectively.

## Who are they interacting with, and how?

These imaging approaches not only provide a census of ‘who is there?,’ they can also reveal physical interactions between polymicrobial community members—such as Mark Welch’s visual demonstration of complex three-dimensional structure in dental plaque communities, with particular taxa preferentially associating with one another [7]—or help understand how far inter-bacterial chemical signals can travel in a community. Baxter et al. discuss their work that reveals the presence of radiating structures of nutrient channels in bacterial colony biofilms [5]. The molecular composition of the extracellular matrix that binds microbes together in biofilm is both an example and a mediator of bacterial interactions. Minero et al.’s article reviews recent work that expands our knowledge of the diversity of nucleic acids present in biofilm matrices, as well as the familiar B-DNA. They and others have demonstrated the presence of both non-canonical DNA structures and RNA [8].

The empirical examples drawn on in these studies result from a range of experimental approaches, from simple *in vitro* microcosms to *in vivo* studies that incorporate consideration of host–microbe interactions and direct observation of microbial communities isolated from natural hosts. With a specific focus on model systems for studying bacterial sociality, Maslowa et al. [9] review the current status of greater wax moth (*Galleria mellonella*) larvae as a powerful halfway house between *in vitro* and *in vivo* studies in mammalian species, drawing on experiments that explore pathogen-pathogen and pathogen-mutualist interactions in this high-throughput model animal host.

Competitive interactions between microbes are common. Bacteria have evolved a remarkable arsenal of protein-based weapons that work to kill or suppress the growth of competitors, and this bacterial weaponry is the focus of the review by Gee and Sharp [10]. In their review, the authors detail the genetic, structural, and key mechanistic differences across major bacterial weaponry systems and highlight the potential of these systems to be exploited therapeutically [10]. Oyama [11] focuses on the broad array of antimicrobial peptides (AMPs) secreted by bacteria to influence competitors at a distance. With the possible exception of bacteriocins, these have been understudied relative to the AMPs produced by animals and plants. Oyama makes the case that until very recently, bacterial AMPs have also been understudied as ecologically important modulators of microbial community interactions, instead viewed one-dimensionally as potential therapeutic leads to be developed outside their natural context.

Munnoch and Hoskisson focus on the nature of interactions within species [12], providing an introduction to the complex division of labour observed in *Streptomyces* populations and how these highly plastic organisms integrate external cues to adopt specialised phenotypes that optimise environmental exploitation and life history decisions. Finally, Randall and Griffin discuss β-lactamases, important antibiotic resistance enzymes that break down β-lactam antibiotics [13]. They address intriguing variation in the social effects of β-lactamases and outline reasons why the protective effects of these enzymes may vary.

## What are the implications of these interactions?

Diverse microbial communities can colonise the intra- or intercellular spaces of plants, as well as the surfaces of their aerial and belowground organs. Escudero-Martínez et al. discuss how these communities and the interactions within them can shape plant growth and health [14]. Bioinoculants are one way we can harness this knowledge of beneficial plant-associated microbes to foster sustainable crop production, and Escudero-Martínez et al. give an expert review of both the challenges and key methodologies in this space. Smith & Holtappels review the role of bacteriophage in shaping the structure and function of these plant-associated microbiomes, including a focus on recent work showing how phage can alter bacterial evolution and impact the composition and diversity of microbial communities [15].

Hall and Baxter also focus on interkingdom interactions, detailing the main bacterial-fungal interactions observed within microbial communities [16]. Their review includes examples of bacterial–fungal antagonism and mutualism, and even interkingdom horizontal gene transfer, within the microbiomes of plants, humans, and the built environment. The impact of bacterial–fungal interactions on health in these various environments is also reviewed. The close relationships between bacteria and fungi within interkingdom communities have been visually confirmed by advanced imaging techniques—both Hall and Baxter and Mark Welch [7] include examples of pathogenic bacteria physically ‘hitch hiking’ on fungal hyphae to penetrate into new host niches, and Baxter et al. contrast the impact of yeast versus hyphal fungal growth on the structure of fungus-bacteria co-cultures at the millimetre scale [5].

Garland et al. focus on the invasion of mobile genetic elements (plasmids) into the microbiome [17]. How plasmids become established and stable within microbial communities, the factors governing this, and the impact of the community and interactions within it on plasmid fate. This acquisition of new plasmids is one way in which the ‘pangenome,’ i.e. the complete genetic content of all strains of a bacterial population (usually defined as a species), can expand. Whelan addresses the following question: What is the effect of ecology on the pangenome [18]? Further to this, Whelan also addresses how the pangenome can be studied within the microbiome using metagenomic sequencing data and, as an expert bioinformatician, outlines current available tools to do this.

Oyama’s review of bacterial AMPs makes the point that studying these molecules through the lens of microbial ecology will enhance our ability to find and develop bacterial AMPs with therapeutic potential [11]. Many AMPs of bacterial, animal, and plant origin are able to kill bacteria or enhance antibiotic action *in vitro*, but very few have demonstrated actual clinical success. It is interesting to note that the only class of AMPs that is in widespread clinical use as antibiotics is of bacterial origin—the polymyxins.

An understanding of bacterial sociality can also be applied to optimise production of bacterial natural products with clinical or industrial value (see the determinants of antimicrobial production by *Streptomyces* species, reviewed by Munnoch and Hoskisson [12]), and to extend the shelf life of antimicrobial natural products in the face of selection for resistance (see the reviews by Randall and Griffin [13] and Garland et al. [17] focussing on β-lactamases and plasmid dynamics, respectively).

Also with an eye to discovering and optimising therapeutics, the potential role of diverse nucleic acid molecules in maintaining biofilm matrix integrity as explored by Minero et al. [8] casts these molecules as targets for novel biofilm-degrading agents. The principle that administering matrix-degrading adjuvants alongside antibiotics to enhance their penetration into biofilms has been demonstrated by the use of enzymes that degrade B-DNA or matrix polysaccharides, but if other molecules are key to matrix function in specific biofilm contexts, then these should instead be targeted. The imaging approaches presented by these authors and Baxter et al. and Bakker and Mark Welch [5–7], are eminently suited to validating the effects of novel therapeutics on biofilms if used with suitable models of pathogenic biofilms.

## Concluding statement

Collectively, the articles in the present special issue highlight some of the breadth and diversity of research topics across bacterial sociality and some of the fundamental methods that are helping us further advance this field. We are grateful to the authors for their contributions and also to the colleagues—including many post-docs and technical professionals—who provided expert peer review for the present special issue.

## Open Access

Open access for this article was enabled by the participation of the University of Warwick in an all-inclusive Read & Publish agreement with Portland Press and the Biochemical Society under an agreement with JISC.

## Abbreviations

AMPs: antimicrobial peptides
CLASI-FISH: combinatorial labelling and spectral imaging - fluorescence in situ hybridisation
